# Direct RNA Sequencing Unfolds the Complex Transcriptome of Vibrio parahaemolyticus

**DOI:** 10.1128/mSystems.00996-21

**Published:** 2021-11-09

**Authors:** Mohamad Al kadi, Eiji Ishii, Dang Tat Truong, Daisuke Motooka, Shigeaki Matsuda, Tetsuya Iida, Toshio Kodama, Daisuke Okuzaki

**Affiliations:** a Department of Bacterial Infections, Research Institute for Microbial Diseases, Osaka University, Osaka, Japan; b Department of Infection Metagenomics, Research Institute for Microbial Diseases, Osaka University, Osaka, Japan; c Bacteriology Department, Institute of Tropical Medicine, Nagasaki University, Nagasaki, Japan; d Single Cell Genomics, Human Immunology, WPI Immunology Frontier Research Center, Osaka University, Osaka, Japan; e Genome Information Research Center, Research Institute for Microbial Diseases, Osaka University, Osaka, Japan; f Institute for Open and Transdisciplinary Research Initiatives, Osaka University, Osaka, Japan; g Center for Infectious Disease Education and Research (CiDER), Osaka University, Osaka, Japan; UCSF

**Keywords:** nanopore, RNA-seq, transcriptome, *Vibrio parahaemolyticus*, pathogenicity islands

## Abstract

Conventional bacterial genome annotation provides information about coding sequences but ignores untranslated regions and operons. However, untranslated regions contain important regulatory elements as well as targets for many regulatory factors, such as small RNAs. Operon maps are also essential for functional gene analysis. In the last decade, considerable progress has been made in the study of bacterial transcriptomes through transcriptome sequencing (RNA-seq). Given the compact nature of bacterial genomes, many challenges still cannot be resolved through short reads generated using classical RNA-seq because of fragmentation and loss of the full-length information. Direct RNA sequencing is a technology that sequences the native RNA directly without information loss or bias. Here, we employed direct RNA sequencing to annotate the Vibrio parahaemolyticus transcriptome with its full features, including transcription start sites (TSSs), transcription termination sites, and operon maps. A total of 4,103 TSSs were identified. In comparison to short-read sequencing, full-length information provided a deeper view of TSS classification, showing that most internal and antisense TSSs were actually a result of gene overlap. Sequencing the transcriptome of V. parahaemolyticus grown with bile allowed us to study the landscape of pathogenicity island Vp-PAI. Some genes in this region were reannotated, providing more accurate annotation to increase precision in their characterization. Quantitative detection of operons in V. parahaemolyticus showed high complexity in some operons, shedding light on a greater extent of regulation within the same operon. Our study using direct RNA sequencing provides a quantitative and high-resolution landscape of the V. parahaemolyticus transcriptome.

**IMPORTANCE** Vibrio parahaemolyticus is a halophilic bacterium found in the marine environment. Outbreaks of gastroenteritis resulting from seafood poisoning by these pathogens have risen over the past 2 decades. Upon ingestion by humans—often through the consumption of raw or undercooked seafood—V. parahaemolyticus senses the host environment and expresses numerous genes, the products of which synergize to synthesize and secrete toxins that can cause acute gastroenteritis. To understand the regulation of such adaptive response, mRNA transcripts must be mapped accurately. However, due to the limitations of common sequencing methods, not all features of bacterial transcriptomes are always reported. We applied direct RNA sequencing to analyze the V. parahaemolyticus transcriptome. Mapping the full features of the transcriptome is anticipated to enhance our understanding of gene regulation in this bacterium and provides a data set for future work. Additionally, this study reveals a deeper view of a complicated transcriptome landscape, demonstrating the importance of applying such methods to other bacterial models.

## INTRODUCTION

Two decades after the first annotation (1), bacterial genomes are still annotated in the same way: an automated pipeline predicts the genes, particularly protein-coding genes, by searching for open reading frames (ORFs) in the genome reference (2). This process ignores other important features included in the genome, such as transcription start sites (TSSs), transcription termination sites (TTSs), small RNAs (sRNAs), and operon maps.

The advent of transcriptome sequencing (RNA-seq) has improved the transcriptome annotation process by combining computational prediction with experimental data (3). This process includes fragmenting RNA, reverse transcription, and sequencing the generated short fragments of cDNA (4). By aligning the generated short reads to the reference genome, these extra features of the transcriptome can be elucidated (5). However, fragmented reads cannot be attributed to their original transcriptional units, which raises many challenges. These include failing to detect operons directly as well as difficulty in differentiating overlapping genes because of the compact nature of bacterial genomes. Many strategies have been developed to overcome such challenges, including detecting TSSs using differential RNA-seq, detecting TTSs using Term-seq (6), and computationally predicting operons from RNA-seq data (3). Recently, long-read sequencing methods have been introduced, allowing full-length transcripts to be sequenced without fragmentation. Direct RNA (dRNA) sequencing from Oxford Nanopore Technologies is the most promising method; it sequences the native RNA directly without major processing, such as fragmentation or reverse transcription, which can introduce artifacts (7).

Vibrio parahaemolyticus is a Gram-negative, halophilic bacterium found in the marine environment. Since 1996, a pandemic strain (O3:K6) has spread around the world, making it one of the leading causes of gastroenteritis and increasing the interest in this bacterium (8). Upon ingestion by humans—often through the consumption of raw or undercooked seafood—V. parahaemolyticus can cause an acute gastroenteritis by expressing various virulence genes. These include thermostable direct hemolysin (TDH) and/or TDH-related hemolysin (TRH), as well as two type 3 secretion systems, T3SS1 and T3SS2. T3SS1 was correlated with mortality in mouse models (9), while T3SS2 is responsible for enterotoxicity (10) and required for intestinal colonization (11).

This study aimed to fully reannotate the V. parahaemolyticus transcriptome under two conditions: growth in Luria-Bertani medium (i) with bile and (ii) without bile, which is a host-derived signal sensed by V. parahaemolyticus to upregulate virulence genes in the intestines (12). Direct RNA sequencing was applied to sequence the native RNA in order to unravel the complexity of the V. parahaemolyticus transcriptome and identify the full features of its annotation, including TSSs, TTSs, and operons.

## RESULTS

### Evaluation of direct RNA sequencing.

Due to the low output of direct RNA sequencing, each sample was sequenced in a separate run on a MinION sequencer. MinION sequencing of the rRNA-depleted polyadenylated native RNA generated 1,361,763 ± 330,638 reads per run on average. The median read length was 744 bp on average, and the mean read quality was 8.6 (see Table S1 at https://github.com/imet-k/DRS/blob/main/Table_S1.docx). A total of 81% ± 4% of the reads were mapped to the genome of Vibrio parahaemolyticus (see Table S2 at https://github.com/imet-k/DRS/blob/main/Table_S2.csv), and almost 2% of the reads were mapped to the RNA calibration strand (RCS) spike-in provided by the manufacturer. None of the reads were mapped to the antisense strand of the RCS. The quality of unmapped reads was lower than that of mapped reads (7 versus 10) (*P* < 0.001) (see Table S2 at the GitHub URL mentioned above). They were shorter, with a median length of 278 bp, compared to 826 bp for mapped reads (*P* < 0.001). Interestingly, when the four runs were compared, a strong correlation (*r* = 0.95, *P* < 0.05) was noticed between the median length of unmapped reads and the mapping rate (see Fig. S1 at https://github.com/imet-k/DRS/blob/main/Fig_S1.tif). Identity was 96% on average for the mapped reads. Reads were also mapped to the transcriptome. Almost 55% of the mapped reads corresponded to the full-length gene (see Fig. S2 at https://github.com/imet-k/DRS/blob/main/Fig_S2.tif). Coverage was uniform, with no bias toward 5′ or 3′ (see Fig. S3 at https://github.com/imet-k/DRS/blob/main/Fig_S3.tif). There was some fragmentation, but it was equally distributed along the gene body, with no bias toward any position. Noise was low due to the lack of bias, and the actual start and end sites were demonstrated by a spike in the frequency of the 5′ or 3′ ends, respectively.

**FIG 1:**
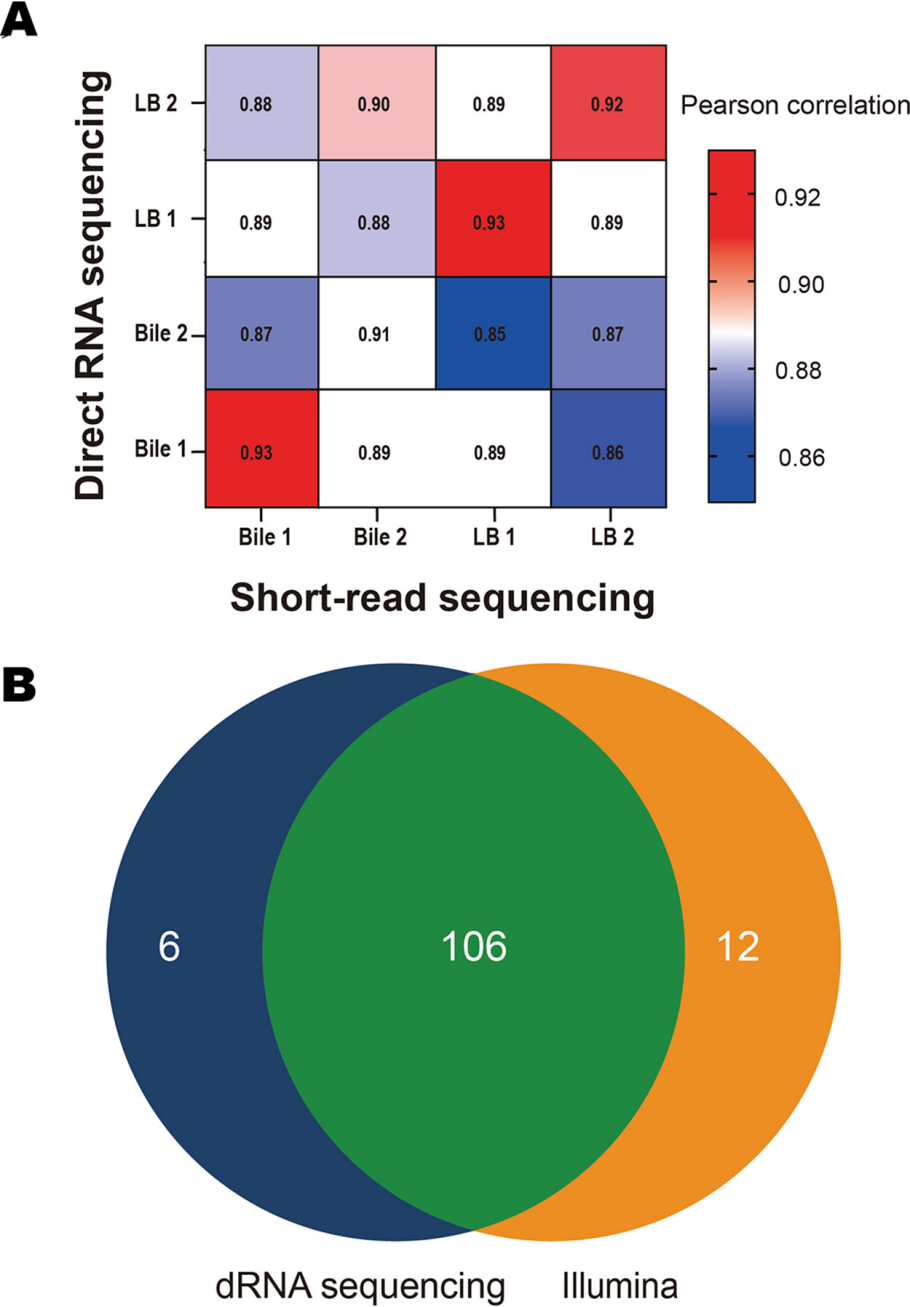
Quantitative evaluation of direct RNA sequencing. (A) Correlation between direct RNA sequencing (dRNA-seq) and short-read Illumina sequencing. A matrix correlation shows a strong correlation between dRNA-seq and Illumina for the same samples, less correlation for biological replicates, and the weakest correlation for different biological samples. (B) Differential gene expression analysis. A Venn diagram illustrates the overlapping of differential gene expression analysis results between dRNA-seq and Illumina. Most of the differentially expressed genes were detected by both methods.

Illumina sequencing was used as the short-read sequencing method to evaluate the output of direct RNA sequencing. Transcripts were quantified using direct RNA sequencing and Illumina reads. Direct RNA sequencing quantification showed a strong correlation with Illumina quantification for the corresponding samples (*r* = 0.93 ± 0.009) (Fig. 1A). This correlation was worse for short genes (<100 bp) (see Fig. S4 at https://github.com/imet-k/DRS/blob/main/Fig_S4.pdf), which was mainly a computational result of false counting the overlapping regions with short neighbor genes in short-read sequencing (see Fig. S5 in the supplemental material). Both methods showed a correlation between biological replicates, indicating reproducibility.

### Differential gene expression analysis.

Differential gene expression analysis was performed using both Illumina and direct RNA sequencing data. In the presence of bile, 118 differentially expressed genes were found with Illumina, and 110 differentially expressed genes were found with direct RNA sequencing (Fig. 1B). Both methods detected the upregulation of the V. parahaemolyticus pathogenicity island (Vp-PAI) on chromosome II. Direct RNA sequencing failed to detect the differential expression of 12 genes that were expressed differentially according to Illumina data. Four genes were small genes that were partially mapped by long reads. The aligner assigned these long reads to the fully covered genes and not to the partially covered small genes (see Fig. S5 in the supplemental material). For the other eight genes, dispersion was apparent between biological replicates that made the fold change statistically insignificant (see Table S3 at https://github.com/imet-k/DRS/blob/main/Table_S3.csv). On the other hand, four genes were found to be differentially expressed by direct RNA sequencing but not by Illumina. Interestingly, one of them was the thermostable direct hemolysin S gene (*tdhS*), whose 3′ end is homologous to that of the other differentially expressed gene, *tdhA* (see Fig. S6 at https://github.com/imet-k/DRS/blob/main/Fig_S6.pdf). As a result, the mapping quality for the reads aligned to the 3′ end of those two genes was poor for short reads and mostly good for long reads.

10.1128/mSystems.00996-21.1 FIG S5 Quantification with short reads leads to overestimation of small genes when their neighbor genes are expressed. In this case, a gene (gene B) partly overlaps a short gene (gene A). The aligner will assign the long reads to their original transcript only (gene B), not to the partly overlapped short gene. On the contrary, short reads would be assigned to the short gene they cover (gene A) as full-length information was lost. Download FIG S5, TIF file, 2.4 MB. Copyright © 2021 Al kadi et al. 2021 Al kadi et al. https://creativecommons.org/licenses/by/4.0/ This content is distributed under the terms of the Creative Commons Attribution 4.0 International license.

### Transcription start site identification.

**FIG 2:**
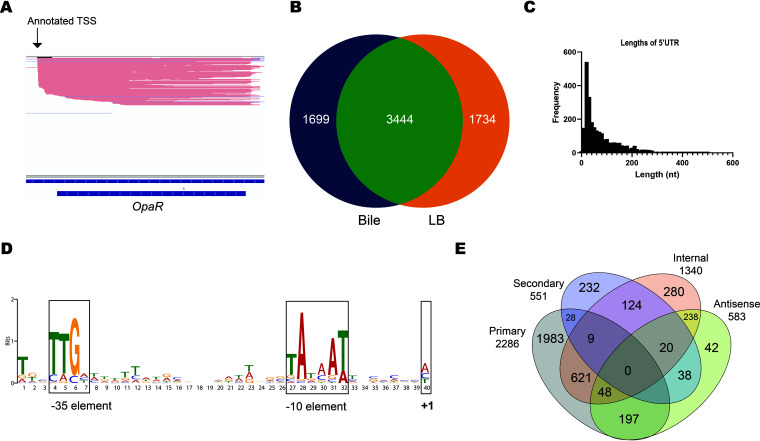
Transcription start site identification. (A) A visual example of direct RNA sequencing reads and an annotated TSS. (B) Detected transcription start sites under both conditions, growing with bile (Bile) and without bile (LB). (C) Distribution of lengths of 5′ UTRs of the annotated genes. (D) Analysis of sequences upstream the transcription start sites showed an enriched motif for the elements of the bacterial σ^70^ promoter. (E) Classification of detected transcription start sites. A TSS can be a (primary) site if it was less than 300 bp from a start codon, an alternative site if it was expressed less than another TSS for the same gene (secondary), internal if it was located in the coding region, or antisense if it was located in the coding region or was less than 100 bp from the coding region on the opposite strand.

Ideally, every long read represents a full-length transcript. Accordingly, TSSs can be obtained easily by clustering the identical 5′ ends of the alignments. However, a quick look at the mapped reads (Fig. 2A) revealed fragmentation, which generated a large number of artifacts. There were 409,346 unique sites out of the total of 1,462,865 alignments. We designed an algorithm (see Materials and Methods) to detect TSSs and eliminate fragmentation noise using two approaches. The first was a transcriptome-aware approach that only considered reads that overlap the start codon with less strict criteria, which generated 3,704 sites (see Materials and Methods). The second was a transcriptome-unaware approach that detected TSSs with stricter criteria while neglecting transcript information, which generated 2,547 sites. Both approaches generated 4,103 sites in total, with 2,148 sites in common. The majority of these sites were located in intergenic regions. On the other hand, the majority of raw clusters were in coding regions (see Fig. S7 in the supplemental material). TSSs were detected under the two conditions with and without bile (Fig. 2B). The same TSSs were detected in at least two replicates for 80% of the expressed genes and in all four replicates for over 50% of expressed genes. To validate these detected TSSs, 22 known start sites were compiled from the literature (see references 40 to 49 below and see Table S4 in the supplemental material), and each of them was compared with the closest TSS in the present data. Most of the sites found in this study were short of the actual TSSs by nine nucleotides on average (see Table S4 in the supplemental material). When the mapped reads were examined, most of them were soft-clipped at the 5′ end, which resulted from the motor protein detachment of the last RNA nucleotides at the 5′ end. The detected TSSs were considered correct if the difference in nucleotide length was less than 14 nucleotides. Accordingly, TSS identification accuracy was 90%.

10.1128/mSystems.00996-21.2 FIG S7 Positions of start sites before and after filtering. Most of the raw sites lie in the coding regions, unlike TSSs obtained by filtering these raw clusters. Download FIG S7, TIF file, 0.1 MB. Copyright © 2021 Al kadi et al. 2021 Al kadi et al. https://creativecommons.org/licenses/by/4.0/ This content is distributed under the terms of the Creative Commons Attribution 4.0 International license.

10.1128/mSystems.00996-21.6 TABLE S4 Comparison of annotated TSSs obtained from literature with detected TSSs. Download Table S4, DOCX file, 0.02 MB. Copyright © 2021 Al kadi et al. 2021 Al kadi et al. https://creativecommons.org/licenses/by/4.0/ This content is distributed under the terms of the Creative Commons Attribution 4.0 International license.

The lengths of the 5′ untranslated regions (5′ UTRs) were calculated, and they peaked at lengths of 10 to 30 nucleotides (Fig. 2C). The regions upstream of the detected TSSs were analyzed by MEME-MOTIF, and the first-ranked motif was the classical −10 element of bacterial promoters. The complete elements of the σ^70^ promoter were also detected, but they were less enriched (Fig. 2D). Additionally, the elements of the σ^54^ promoter were also enriched (see Fig. S8 at https://github.com/imet-k/DRS/blob/main/Fig_S8.pdf). Their motifs were detected for genes involved in flagellar assembly, transportation, and metabolism.

The detected TSSs were classified into five categories (Fig. 2E). Primary TSSs (pTSSs) are the main transcription sites for annotated genes, secondary TSSs (sTSSs) are alternative transcription start sites for annotated genes with lower expression, internal TSSs (iTSSs) are the sites that start within the coding region, antisense TSSs (asTSSs) are the sites that start within the coding region on the opposite strand or within ±100 bp of the gene-coding region, and orphan TSSs (oTSSs) are not close to any annotated gene (over 300 bp away from the closest gene). In many cases, a TSS can be categorized into multiple categories. For example, a TSS that exists in the coding region on the opposite strand was categorized as both iTSS and asTSS. To obtain a deeper view of these TSSs, end sites were also identified. A total of 2,886 primary sites were found, with 1,983 exclusively primary, 678 internal, 245 antisense, and 37 secondary TSSs. Primary-internal TSSs mostly resulted from the overlapping of the 5′ UTR with the coding region of an upstream gene, as most of them were found in the 3′ end of a previous gene body and ended in the 3′ UTR of the gene of interest. Similarly, antisense pTSSs mostly resulted from the 5′ UTR overlapping a neighbor gene on the opposite strand. This was evidenced by the fact that most primary-antisense sites were found near the 5′ end of a neighbor gene on the opposite strand and ended at the 3′ end of the gene of interest. Finally, most of the primary-secondary TSSs occurred in operons. For example, *yfcE* is transcribed both individually and in an operon with the upstream gene *VP_RS23530* (see Fig. S9 in the supplemental material). Thus, the TSS for *yfcE-VP_RS23530* is primary for the gene *VP_RS23530* and secondary for *yfcE*. A total of 551 secondary TSSs were found, which were distributed in similar proportions to those of the pTSSs. Most of them were exclusively secondary sites (332 sites), and the rest were secondary-internal sites (153 sites), secondary-antisense sites (58 sites), and secondary-primary sites (37 sites). A total of 1,340 internal TSSs were found, but only 280 sites were exclusively internal. One-third of iTSSs were in the first 2% of the gene body at the 5′ end, signifying possible leaderless genes, and the rest were distributed equally over the gene body. There were 583 antisense TSSs, which were defined as TSSs occurring on the opposite strand of the coding region or in the UTR regions (less than 100 bp from the start codon). Most of the asTSSs occurring in the untranslated region were also pTSSs or sTSSs for a neighbor gene, suggesting a high number of bidirectional promoters. On the other hand, most of the remaining asTSSs were internal on the opposite strand to the coding region, suggesting a regulatory role. Internal asTSSs were expressed at considerably higher levels than the rest.

10.1128/mSystems.00996-21.3 FIG S9 Detected transcription start sites for *yfcE*. Two TSSs were detected for *yfcE*: a TSS with high expression before the start codon and a TSS with low expression that starts before the upstream gene. Download FIG S9, TIF file, 1.6 MB. Copyright © 2021 Al kadi et al. 2021 Al kadi et al. https://creativecommons.org/licenses/by/4.0/ This content is distributed under the terms of the Creative Commons Attribution 4.0 International license.

### Transcription termination site identification.

**FIG 3:**
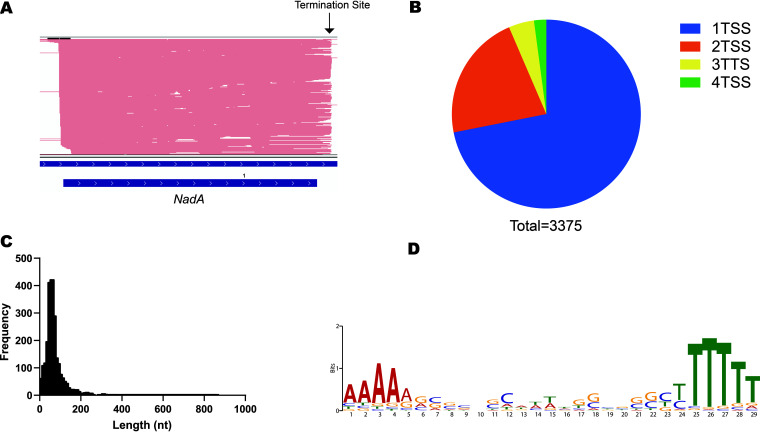
Transcription termination site identification. (A) An example of a detected TTS. The TTS was selected as the most frequent end site among the end sites after the stop codon. (B) Number of TTSs per gene. Most genes had only one termination site, but others had up to 4 sites. (C) Distribution of lengths of 3′ UTRs for annotated genes. (D) Detection of the rho-independent motif by analyzing the sequences at the termination site.

Sequencing the full-length RNA allowed the identification of the 3′ ends of transcripts, genes, and operons. TTSs were calculated by clustering the read ends, and the site with the highest frequency was identified as the termination site. This is because some transcripts read through the termination site (Fig. 3A). A total of 4,613 termination sites were detected for 3,375 genes. The majority (71%) showed a single termination site, and the rest had two or more sites per gene (Fig. 3B). For 40% of these genes, multiple sites corresponded to the main operon termination site, in addition to other termination sites inside the operon that generated suboperons. The rest (∼60%) of the genes with multiple TTSs were alternative sites for the same transcriptional unit, with a distance of approximately 50 bp between these alternative sites. The 3′-UTR lengths were calculated, and most of them were less than 100 bp (Fig. 3C). Motif analysis of the sequences around the termination sites showed an enriched rho-independent motif (Fig. 3D).

The majority of genes (67%) ended in the intergenic region, while the rest ended in the coding region of a neighboring gene regardless of the strand direction.

### Operons.

**FIG 4:**
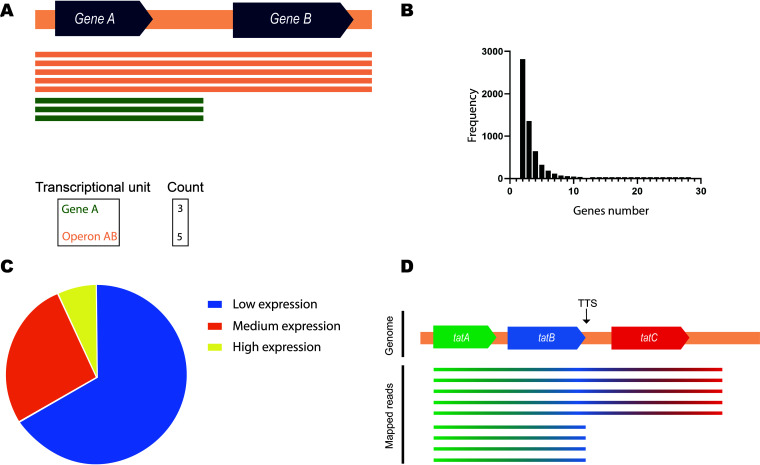
Operon detection. (A) Operon detection and quantification. Operons can be detected using long reads easily in both qualitative and quantitative ways. (B) Distribution of operon gene numbers. (C) Expression level of detected operons relative to their genes’ expression. Operons have low (<20%), medium (20 to 60%) or high (>60%) expression compared to their genes’ expression. (D) Suboperon in the *tatABC* operon. *tatABC* showed two operons, *tatABC* and *tatAB*.

Long-read sequencing allowed direct and simple detection of operons by clustering reads that intersect more than two genes on the same strand (Fig. 4A). A total of 5,836 operons were detected, covering 3,865 genes out of the 4,686 total expressed genes. The distance between genes in the same operon was 56 ± 0.8 bp on average. The majority of the detected operons had two genes (Fig. 4B), but some of them had up to 28 genes. Due to the full-length information, the detected operons were quantified. Unlike short-read sequencing, reads could be attributed to their corresponding transcriptional unit (Fig. 4A), allowing quantitative analysis for each of the detected operons. In numerous cases, genes were transcribed alternatively in more than one operon, where the gene would be transcribed mainly in one operon and to a less extent in other alternative operons. Therefore, operons were classified as having low, medium, or high expression based on their expression relative to that of their composing genes: <20%, 20 to 60%, and >60%, respectively. In other words, high expression of an operon means that one of its genes is transcribed primarily in this operon. Computationally, it means that for at least one of the genes, more than 60% of its gene expression is attributed to this operon. Low expression of an operon means all of its genes are transcribed secondarily in this operon, and their primary transcription produces other transcriptional units (i.e., less than 20% of the gene expression for each of the operon’s genes is attributed to transcriptional units other than this operon). Accordingly, two-thirds of the detected operons (67%) had low expression relative to the genes composing them, and only around 7% were dominantly expressed (Fig. 4C).

However, this still does not show the big picture. The operon was considered dominantly expressed if at least one gene was expressed mainly in this operon. This does not necessarily apply to the other genes in the operon. For example, some regions, such as *tatABC* genes, showed complicated operon landscapes (Fig. 4D) in which a termination site generated a suboperon (*tatAB*) in addition to the termination site of the main operon (*tatABC*). Analyzing the sequence of *tatABC* region predicted the same two rho-independent terminators detected by our data. Thus, *tatC* is transcribed mainly in one operon, *tatABC*, while *tatA and tatB* genes are transcribed in two operons. Another case is *VP2784–VP2783*, in which the first gene, *VP2784*, is transcribed mainly in the operon *VP2784–VP2783*, but only 14% of the reads that were mapped to VP2783 were attributed to this operon. The rest were transcribed from a separate TSS for the monocistronic transcript *VP2783*. Similarly, many of the genes that were reported to be a part of polycistronic mRNA were mainly monocistronic—that is, transcribed individually—with some readthrough transcription that extended to the next gene/genes. Of the 3,865 genes reported to be part of an operon in our study, ∼20% were actually transcribed individually at a level of 90% or more as monocistronic RNA with low coexpression with the neighboring genes. The expression of detected operons was reported relative to the genes that composed them (see Data Set S1 in the supplemental material).

10.1128/mSystems.00996-21.7 DATA SET S1 Operons detected over four samples. Download Data Set S1, XLSX file, 0.4 MB. Copyright © 2021 Al kadi et al. 2021 Al kadi et al. https://creativecommons.org/licenses/by/4.0/ This content is distributed under the terms of the Creative Commons Attribution 4.0 International license.

### Analyzing the transcriptional landscape of the pathogenicity island.

**FIG 5:**
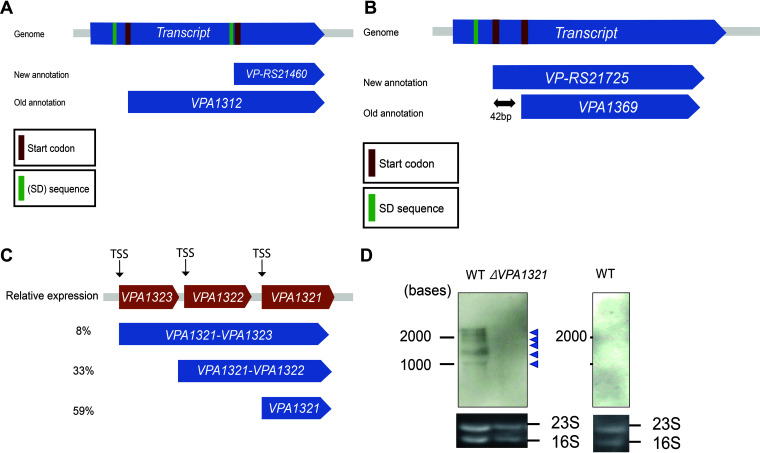
Analysis of the transcriptional landscape of the pathogenicity island. (A) Reannotation of the *VP-RS21460* gene; our reconstructed transcript supports the old annotation, *VPA1312*. It covers the gene body and has a Shine-Dalgarno (SD) sequence near the start codon. (B) Reannotation of the *VPA1369* gene. Our reconstructed transcript supports the new annotation, *VP-RS21725*. It covers the gene body and has a Shine-Dalgarno (SD) sequence near the start codon. (C) Suboperons in the operon *VPA1321–VPA1323*; all transcriptional units share the same transcription termination sites, but each of them has its own transcription start site (TSS). (D) Northern blot of VPA1321 in Vibrio parahaemolyticus in WT and Δ*VPA1321* strains growing with bile and an NaCl concentration of 0.5% (left) and a WT strain growing without bile and an NaCl concentration of 3% (right).

Growing V. parahaemolyticus with bile induced the expression of most of the genes contained in the pathogenicity island Vp-PAI, including several T3SS2 genes (see Data Set S2 in the supplemental material). This allowed us to annotate and analyze the transcriptional landscape of this region. In order to enhance the quality of RefSeq (the Reference Sequence) genomes, the RefSeq project at the National Center for Biotechnology Information (NCBI) reannotated prokaryotic genomes with an updated pipeline, PGAP (Prokaryotic Genome Annotation Pipeline), including different data and algorithms (13). As a result, V. parahaemolyticus annotation in RefSeq was changed drastically, including deleting and adding some genes and changing ORFs for others. In the pathogenicity island Vp-PAI, 19 genes were deleted from the old annotation, two genes were added, 15 genes had their ORFs changed, and 50 genes stayed unchanged. We used direct RNA sequencing data to reannotate the genes in Vp-PAI island and assign them to the correct annotation based on our data (see Data Set S2 in the supplemental material). Some ORFs changed significantly in the new annotation, such as *VPA1312*, which was replaced by a new gene, *VP_RS21460*, with a shorter ORF of 209 bp instead of the old 524-bp ORF. Our data support the old annotation, with the reconstructed transcript covering the whole length of its ORF and containing a Shine-Dalgarno (SD) sequence near the start codon (Fig. 5A).

10.1128/mSystems.00996-21.8 DATA SET S2 Pathogenicity island reannotation. Download Data Set S2, XLSX file, 0.01 MB. Copyright © 2021 Al kadi et al. 2021 Al kadi et al. https://creativecommons.org/licenses/by/4.0/ This content is distributed under the terms of the Creative Commons Attribution 4.0 International license.

In other instances, the new annotation was more accurate in annotating genes, such as *VPA1369*. The new counterpart gene, VP-*RS21725*, is 42 bp longer, and our reconstructed transcript supports this annotation. It covers the new annotated ORF and includes an SD sequence (Fig. 5B). Most deleted genes have short ORFs with products of unknown functions and low expression, justifying dismissing them as functional coding genes.

In addition, we discovered three novel intergenic noncoding RNAs that were upregulated while growing with bile, suggesting a possible role in pathogenicity given their position in the pathogenicity island (see Table S4 in the supplemental material). Finally, operons in the pathogenicity island showed a complicated structure as in the rest of the genome. For example, the *VPA1321*, *VPA1322*, and *VPA1323* genes share the same termination site, but each of them has its own TSS, creating three transcriptional units, including two operons, *VPA1321–VPA1323* and *VPA1321–VPA1322* (Fig. 5C). Northern blot analysis was performed using a probe for the common *VPA1321* gene. All aforementioned transcriptional units were detected (see Fig. S10 in the supplemental material). Moreover, two additional bands were detected. Direct RNA sequencing also detected these products as alternative TSS products for *VPA1321* and *VPA1322* (Fig. 5D). Specificity was confirmed by the lack of bands in the deletion mutant (Δ*VPA1321*) and the wild-type strain grown under noninductive conditions (high salt without bile). Northern blot analysis was performed using a probe for *VPA1323*. We detected a band with the same size (∼2,200 bases) as the band detected by the *VPA1321* probe in the wild-type strain grown with bile (see Fig. S11 in the supplemental material).

10.1128/mSystems.00996-21.4 FIG S10 Direct RNA sequencing detected transcripts for the *VPA1321–VPA1323* genes. Download FIG S10, PDF file, 0.8 MB. Copyright © 2021 Al kadi et al. 2021 Al kadi et al. https://creativecommons.org/licenses/by/4.0/ This content is distributed under the terms of the Creative Commons Attribution 4.0 International license.

10.1128/mSystems.00996-21.5 FIG S11 Northern blot analysis with a probe for VPA1323. Download FIG S11, PDF file, 0.10 MB. Copyright © 2021 Al kadi et al. 2021 Al kadi et al. https://creativecommons.org/licenses/by/4.0/ This content is distributed under the terms of the Creative Commons Attribution 4.0 International license.

Interestingly, excludons (i.e., antisense transcription generated by an operon extending to the neighbor genes on the opposite strand) were detected in the bile condition. Most notably, suboperons *VPA1337–VPA1334* and *VPA1335–VPA1334* overlap *VPA1332* on the opposite strand, the master regulator of T3SS2.

## DISCUSSION

The quantification of direct RNA sequencing reads demonstrated reliability and reproducibility. Differential gene expression analysis results conformed with those of Illumina. However, sequencing the samples in different runs seems to increase the variance between samples. On the other hand, direct RNA sequencing was better for the quantification of homologous genes such as *tdhS* and *tdhA*. Both methods detected the upregulation of T3SS2 genes in accordance with other transcriptome analyses, but there was a significant difference in detected differentially expressed genes (14, 15) (see Data Set S3 in the supplemental material). This is might be because of the difference in incubation time with bile—30 min versus 3 h in previous studies and our study, respectively.

10.1128/mSystems.00996-21.9 DATA SET S3 Differential gene expression results. Download Data Set S3, CSV file, 0.5 MB. Copyright © 2021 Al kadi et al. 2021 Al kadi et al. https://creativecommons.org/licenses/by/4.0/ This content is distributed under the terms of the Creative Commons Attribution 4.0 International license.

Two obstacles were involved in the identification of TSSs. First, fragmentation generated a large number of false start sites. However, because fragmentation was random and not biased, it was possible to find true TSSs by looking for significant spikes in start sites and ignoring other start sites. This process was automated, making TSS identification easy and fast. Compared with known TSSs obtained from the literature, the automated pipeline results agreed with the visual assessment. One drawback of nanopore direct RNA sequencing is the inability to sequence the last 15 nucleotides (16). This phenomenon was also observed in the present study, in which ∼9 nucleotides were missing on average. Variation was random, even for the same TSS across biological replicates. Thus, it was not possible to correct it by adding a specific number of nucleotides to compensate for the missing nucleotides, as was suggested previously (16).

With no database for V. parahaemolyticus TSSs, individually identified TSSs were obtained from the literature to validate the present results. The pipeline correctly identified 20 known TSSs but not at a single-nucleotide resolution. Two genes were not identified correctly, which might be due to the difference in growth conditions. Thus, a database for V. parahaemolyticus TSSs was constructed in this study (see Data Set S4 in the supplemental material).

10.1128/mSystems.00996-21.10 DATA SET S4 Detected transcription start sites. Download Data Set S4, XLSX file, 0.5 MB. Copyright © 2021 Al kadi et al. 2021 Al kadi et al. https://creativecommons.org/licenses/by/4.0/ This content is distributed under the terms of the Creative Commons Attribution 4.0 International license.

Analyzing the sequences upstream of the detected TSSs revealed the expected classical elements of the σ^70^ recognition sequence, in accordance with other *Vibrio* species studies (17, 18). The −35 element was successfully detected, while a previous study that used nanopore direct RNA sequencing failed to detect the −35 element in Escherichia coli (16). This may be a result of using different strategies in identifying transcription start sites. Because of fragmentation, we consider the first site in a cluster as the TSS and the rest as truncated reads, unlike Grünberger et al. (16), who chose the median site as the TSS.

The promoter of RpoN (σ^54^) was detected in 61 genes/operons. RpoN is an alternative sigma factor that is involved in metabolism (19), flagellar biosynthesis (20), and virulence (21). Similarly, recognition sequences were found for RpoN in flagellar synthesis, metabolism, and transportation genes, showing the potential of our study in identifying genes that are regulated by RpoN.

This study allowed a deeper analysis of TSS classification because of the availability of transcript end information for these TSSs. For example, from such information, it can be surmised that internal pTSSs were mostly internal as a result of untranslated regions overlapping between two genes. Full-length information provides a more comprehensive view of TSS classes, as opposed to short-read sequencing, which classifies TSSs as points in the genome. The results for V. parahaemolyticus TSSs repartition were similar to those of the closely related species Vibrio cholerae (17) for the internal, secondary, and orphan classes (24% versus 24%, 10% versus 8% and 3% versus 4%, respectively). However, the primary and antisense proportions were highly different (52% versus 22% and 10% versus 41%, respectively). Widespread antisense transcription has been reported in prokaryotes (22), including enteric bacteria (23). Although the present results showed relatively fewer antisense TSSs than those of V. cholerae, a low proportion of antisense TSSs has been reported in the closely related species Vibrio harveyi (12 to 17%) (18) and in Salmonella enterica (13%) (24). One possible reason for the low asTSS proportion could be that many of the antisense RNA (asRNA) transcripts were so short that they were excluded from the present experiment (see Materials and Methods). The number of small asRNAs was also low in another study carried out in the closely related species V. cholerae (25). Antisense transcript expression was found to be low (23), which was also the case in the present study. Thus, the low asTSS proportion in this study could result from the inability to detect asTSSs expressed at a low level. However, this still does not explain the low abundance of asTSSs in V. harveyi (18) because both previous studies used the same protocol. Low expression of many of these asTSSs may suggests that they are merely transcription noise, as suggested before (23), but highly expressed asRNAs still have a potential regulatory role that warrants further investigation.

TTS identification using second-generation RNA-seq is a challenging task in bacteria as a consequence of bacterial transcripts lacking a poly(A) tail (6). Using direct RNA sequencing, the termination sites for 3,375 genes were identified simply by clustering the read ends that were mapped to coding genes (see Data Set 5 at https://github.com/imet-k/DRS/blob/main/Dataset5_TTSs.xlsx). An analysis of the sequences around the termination sites showed a significant number of rho-independent motifs (Fig. 3D). 3′ UTRs, like Rho-independent terminators, are important regulatory elements in bacteria. Different 3′-UTR sequences and lengths affect the expression of their mRNA (26, 27). The 3′ UTR is also considered a reservoir for regulatory small RNAs (sRNAs) (28). In this study, 3′ UTRs were mapped on a wide scale for V. parahaemolyticus, which offers a rich database to mine for regulatory elements and targets.

Operon identification on a wide scale depends on prediction rather than direct detection (3). Recently, long-read RNA sequencing has allowed the detection of operons directly without the need for computational prediction (29). By simply looking for the genes that intersect with direct RNA sequencing (dRNA-seq) long reads, operons containing up to 28 genes were detected. Operons were reported qualitatively and quantitatively. Moreover, we report the occurrence of numerous suboperons, which are transcriptional units independently transcribed from the main TSS in the operon (30). The present results revealed a complex landscape for some operons, in which many suboperons exist within the same operon. For example, we found a suboperon in the *tatABC* operon (i.e., *tatAB* in the *tatABC*), which was unreported in previous study in *Vibrio* spp. (31). This modularity can have significant implications on the function of the system encoded by this operon. Genes in the same operon undergo the same regulation and are functionally related. Thus, discovering suboperons in operons can offer a more detailed view of the functional relationships between its genes. This modularity was also present in the pathogenic island. For example, the case of VPA1312–VPA1323 suggests a functional dependency of VPA1322 and VPA1323 on VPA1321, since they are only expressed along with VPA1321 in the same transcriptional unit. However, since the main bulk of VPA1321 transcription comes from its own TSS, the opposite might not be true.

Readthrough could extend some operons to create longer operons at lower expression levels. Our results showed a significant number of these extended operons. Although they can be thought of as by-products, it has been reported that readthrough can significantly affect the expression of downstream genes under specific conditions (32). Mapping these extended operons globally has the potential to uncover such regulation.

In conclusion, utilizing direct RNA sequencing, we generated a map of the Vibrio parahaemolyticus transcriptome, including transcription start and termination sites. Furthermore, full-length information provided a more comprehensive view of TSSs and TTSs. A complex landscape was revealed for the Vibrio parahaemolyticus transcriptome, in which operons are not always just a single transcriptional unit but can consist of many suboperons with various expression levels, thereby raising transcriptional regulation to a higher level. Additionally, the pathogenic island Vp-PAI annotation was corrected to provide an accurate annotation that enables researchers to better study this island.

## MATERIALS AND METHODS

### RNA isolation.

Vibrio parahaemolyticus RIMD2210633 (KP-positive, serotype O3:K6) was obtained from the Pathogenic Microbes Repository Unit, International Research Center for Infectious Diseases, Research Institute for Microbial Diseases, Osaka University, Osaka, Japan, and was used for RNA isolation. V. parahaemolyticus cells were grown in Luria-Bertani broth containing 0.5% NaCl at 37°C with or without bile acids (0.4%) (Sigma, USA) for 3 h without shaking. Two biological replicates were used for each condition. RNAprotect bacterial reagent was added to the culture to stabilize the RNA, and total RNA was extracted using a silica membrane column of the RNeasy minikit according to the manufacturer’s instructions (Qiagen, USA). rRNA was depleted from total RNA using the Ribo-Zero rRNA removal kit (Illumina, USA). Nanopore direct RNA sequencing library preparation requires poly(A) RNA. Thus, depleted RNA was polyadenylated using E. coli poly(A) polymerase (Bio England Labs) to add poly(A) tails. Poly adenylated RNA was purified using the Agencourt AMPure XP kit (Beckman Coulter, USA). The quality and quantity of RNA were measured using an Agilent Bioanalyzer 2100 (Agilent Technologies, USA) and Nanodrop spectrophotometer (Life Technologies, USA), Qubit RNA HS assay kit (Life Technologies, USA), and Qubit 2.0 fluorometer (Life Technologies).

### RNA sequencing.

Illumina libraries were prepared using a TruSeq stranded total RNA kit (Illumina, USA) following the manufacturer’s instructions and sequenced on a HiSeq 3000 sequencer (Illumina).

A direct RNA sequencing library was prepared using a direct RNA sequencing kit (SQK-RNA001; Oxford Nanopore Technologies) from approximately 500 ng of poly(A) RNA input following the manufacturer’s instructions. Briefly, a reverse transcription adapter was ligated using T4 DNA ligase (New England Biolabs), and first-strand cDNA was synthesized using SuperScript III reverse transcriptase (Thermo Fisher Scientific). Sequencing adapters were ligated using T4 DNA ligase (New England Biolabs). The library was loaded onto a MinION flow cell and sequenced for 48 h. The RNA calibration strand (RCS) spike-in was added to the run in the first step as a control.

### Northern blot.

Probes were synthesized using PCR digoxigenin (DIG) probe synthesis kit (Roche, Germany). For *VPA1321*, the following primers were used: 5′-AGTTCGTGCCGCTCAACTAA-3′ and 5′-AGACAGCGCACCATTTGAGA-3′. For *VPA1323*, the following primers were used: 5′-TGATTATAACAACGGGCCTGA-3′ and 5′-TTGTAGCCTACCCCATTATCAAA-3′. Deletion mutant Δ *VPA1321* was used as a negative control (33).

V. parahaemolyticus cells were grown at 37°C in Luria-Bertani broth containing 0.5% NaCl with bile acids (0.4%) (Sigma, USA) or in Luria-Bertani broth containing 3% NaCl until they reached an optical density at 600 nm (OD_600_) of 0.6. RNA extraction was performed using hot acidic phenol RNA isolation (34). Two milliliters of culture was centrifuged at 12,000 rpm for 2 min, and supernatant was removed. Bacterial cells were lysed with lysis buffer, and acidic phenol was added. After being heated at 65°C for 5 min, the mixture was centrifuged, and the upper aqueous layer was recovered. RNA was precipitated, washed with ethanol, and dried. Nuclease-free water was added, and the concentration was calculated by Nanodrop spectrophotometer (Life Technologies, USA). Denaturing agarose gel electrophoresis was performed using 1 μg of RNA. rRNA was visualized to check RNA integrity.

RNA was transferred to a positively charged nylon membrane (Roche, Germany) and immobilized using UV radiation. Hybridization was performed at 50°C for 4 h in Easy Hyb buffer (Roche, Germany). The membrane was washed with high-stringency buffer and blocked with blocking solution containing blocking reagent (Roche, Germany). Anti-digoxigenin-alkaline phosphatase (DIG-AP) (Roche, Germany) was added to the membrane, and the mixture was incubated for 30 min. Finally, the membrane was equilibrated and a chemiluminescent substrate was added, CDP-STAR (Roche, Germany). The membrane was exposed to Amersham Hyperfilm ECL (GE Healthcare, USA).

### Quantification and differential gene expression analysis.

Reads were aligned against the Vibrio parahaemolyticus RIMD 2210633 reference transcriptome (assembly GCF_000196095.1) using Minimap2, with short- and long-read parameters for Illumina and MinION reads, respectively. The alignment file was converted to the more concise format bed file using bedtools (35). The first column, scaffold, is actually the gene that the read is mapped to. Using a simple code written in python, these scaffolds were counted (https://github.com/imet-k/DRS/blob/main/count_from_bed.py). Reads per kilobase per million (RPKM) were calculated for Illumina data, and reads per million (RPM) were calculated for direct RNA sequencing. The correlation between samples was measured with the Spearman rank correlation coefficient using RPKM and RPM for Illumina and direct RNA sequencing, respectively.

Counts were modeled using the DESeq2 tool (36) for both data sets (Illumina and direct RNA sequencing) following negative binominal distribution, and differential gene expression analysis was performed between the two biological samples using the iDEP web application (37). Genes with a log_2_ fold change greater than 2 and a *P* value of <0.05 were considered significantly differentially expressed.

### Direct RNA sequencing evaluation.

Reads were aligned against the Vibrio parahaemolyticus RIMD 2210633 reference genome (assembly GCF_000196095.1) and the control RNA (ENO2 gene) using Minimap2. The mapping rate, quality, and read lengths were calculated from the alignment file using custom code.

### Transcription start site identification.

TSSs were identified using two approaches: transcriptome awareness and unawareness. The sites detected from both approaches were combined and reported after removing the duplicates. The alignment file was converted to a bed file, a more concise form, using bedtools (35). We wrote a simple code in python to process the generated bed file and detect the transcription start sites (https://github.com/imet-k/DRS/blob/main/TSS.py). In the transcriptome-unaware approach, start sites that were on the same strand and close to each other (less than 10 bp apart) were clustered together, and the most extreme site was reported as the start site, assuming that the rest of the reads were shorter because of fragmentation and/or missing bases at the 5′ end (see Discussion). Coverage at each position in the genome was calculated using bedtools (35), and start sites were filtered based on the base coverage at each site. In the transcriptome-aware approach, start codon information was provided. Only reads that overlapped the start codons were considered. These reads were clustered for every start codon and filtered based on the total number of reads that overlapped the start codon.

For *VPA1321–VPA1323* transcript detection, the transcriptome-unaware approach was used with less stringent criteria: no coverage comparison, with a 235-bp clustering window instead of a 10-bp window.

### Transcription termination site identification.

Similar to TSSs, only reads that overlapped the stop codon were considered. To obtain overlapped reads, we used the bedtools intersect tool and processed the generated file using a simple code written in python (https://github.com/imet-k/DRS/blob/main/TTS_identification.py). End sites were clustered. End sites have a greater chance of being the actual site because they are not missed by the sequencer and are relatively protected by the poly(A) tail. In addition, termination can sometimes be weak such that transcription will continue through the termination site (readthrough). Therefore, the most frequent site in the cluster was reported as the TTS.

### Operon detection.

The alignment file was converted to a bed file, which is more concise. Using bedtools (35), intersections between reads and genes were obtained. Reads that intersected more than two genes were considered if they were on the same strand and had sufficient mapping quality (≥60). Identical operons were clustered together and reported along with their count and compromising genes. To measure the relative expression of each operon, the operon/total gene count ratio was reported for every operon.

### Motif detection.

Sequences to be analyzed were extracted as fasta files using a custom script. MEME-SUITE tool (38) was used to analyze these sequences. Where the fasta file contained more than 1,000 sequences, it was split into files with 999 sequences at most.

### Statistical analysis.

The mean and standard deviation were used for data analysis. A two-tailed *t* test was used for statistical analysis. *P* < 0.05 was considered statistically significant. Correlation was measured using the Pearson coefficient. Statistical analysis was performed using scipy.stats package in python.

### Data availability.

The data sets that support the conclusions of this article are available in the DDBJ database under the accession number PRJNA775855.
