# Supplementary figures and images for: Direct RNA Sequencing Unfolds the Complex Transcriptome of Vibrio parahaemolyticus
Source: mSystems. 2021 Nov 9;6(6):e00996-21. doi: 10.1128/mSystems.00996-21 (PMC8577284; doi:10.1128/mSystems.00996-21)

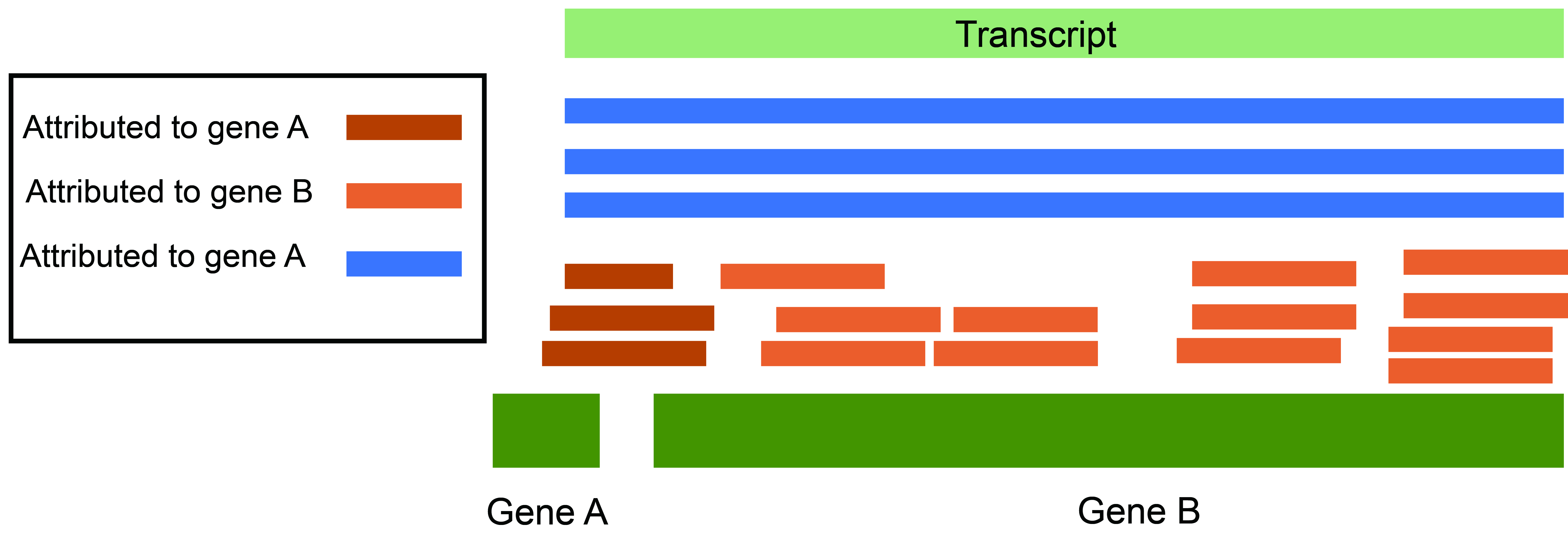

Supplement: FIG S5 [file msystems.00996-21-sf005.tif]

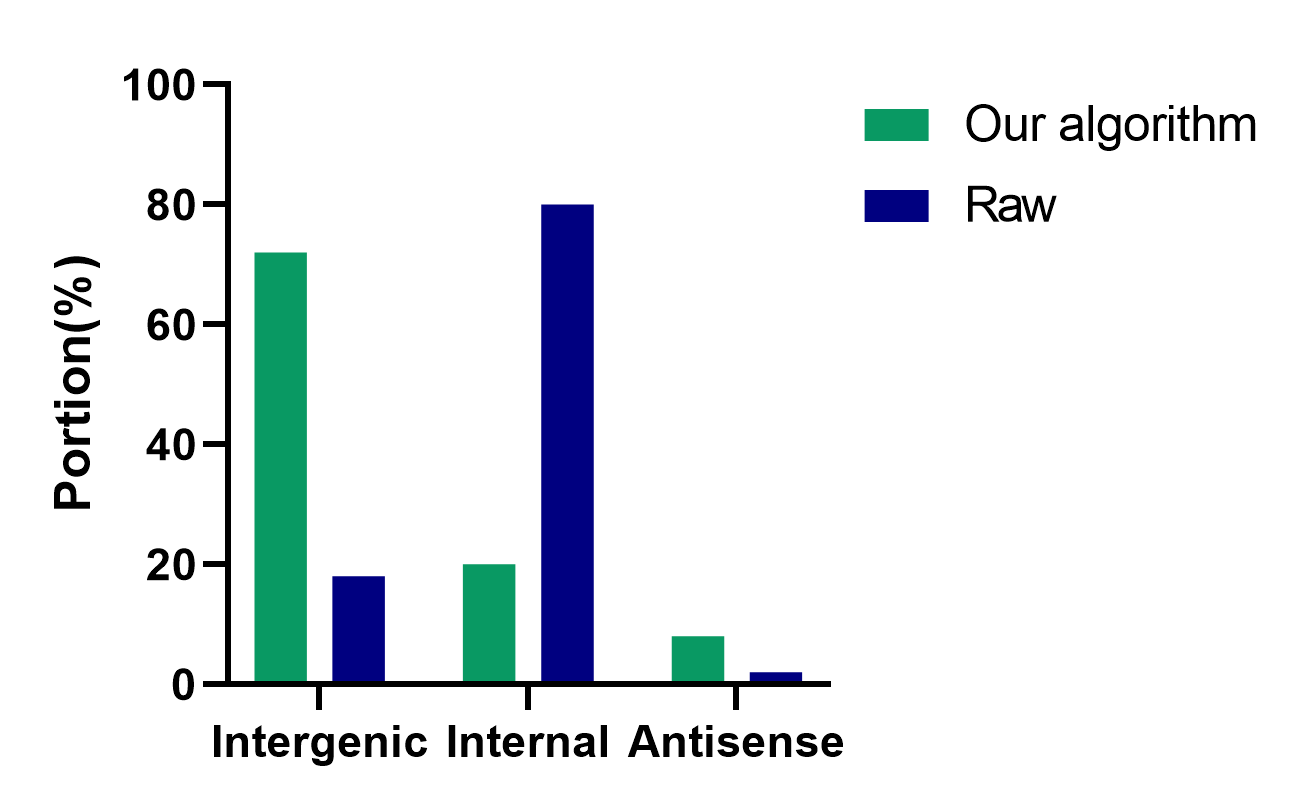

Supplement: FIG S7 [file msystems.00996-21-sf007.tif]

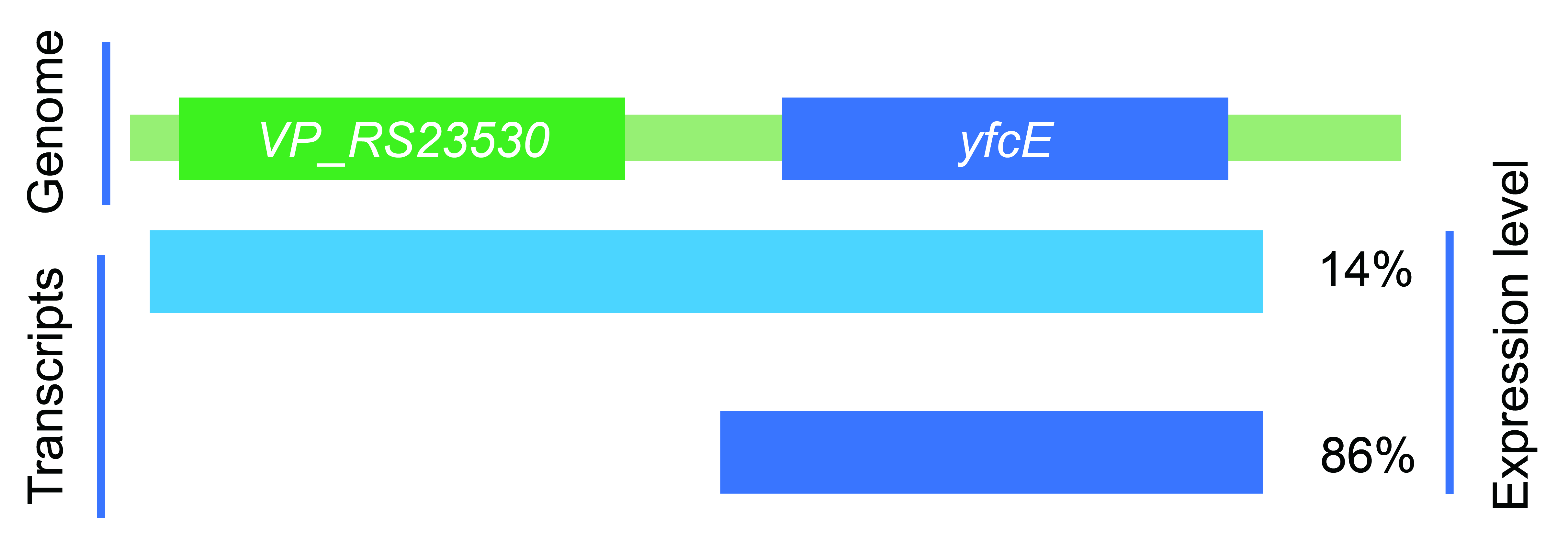

Supplement: FIG S9 [file msystems.00996-21-sf009.tif]

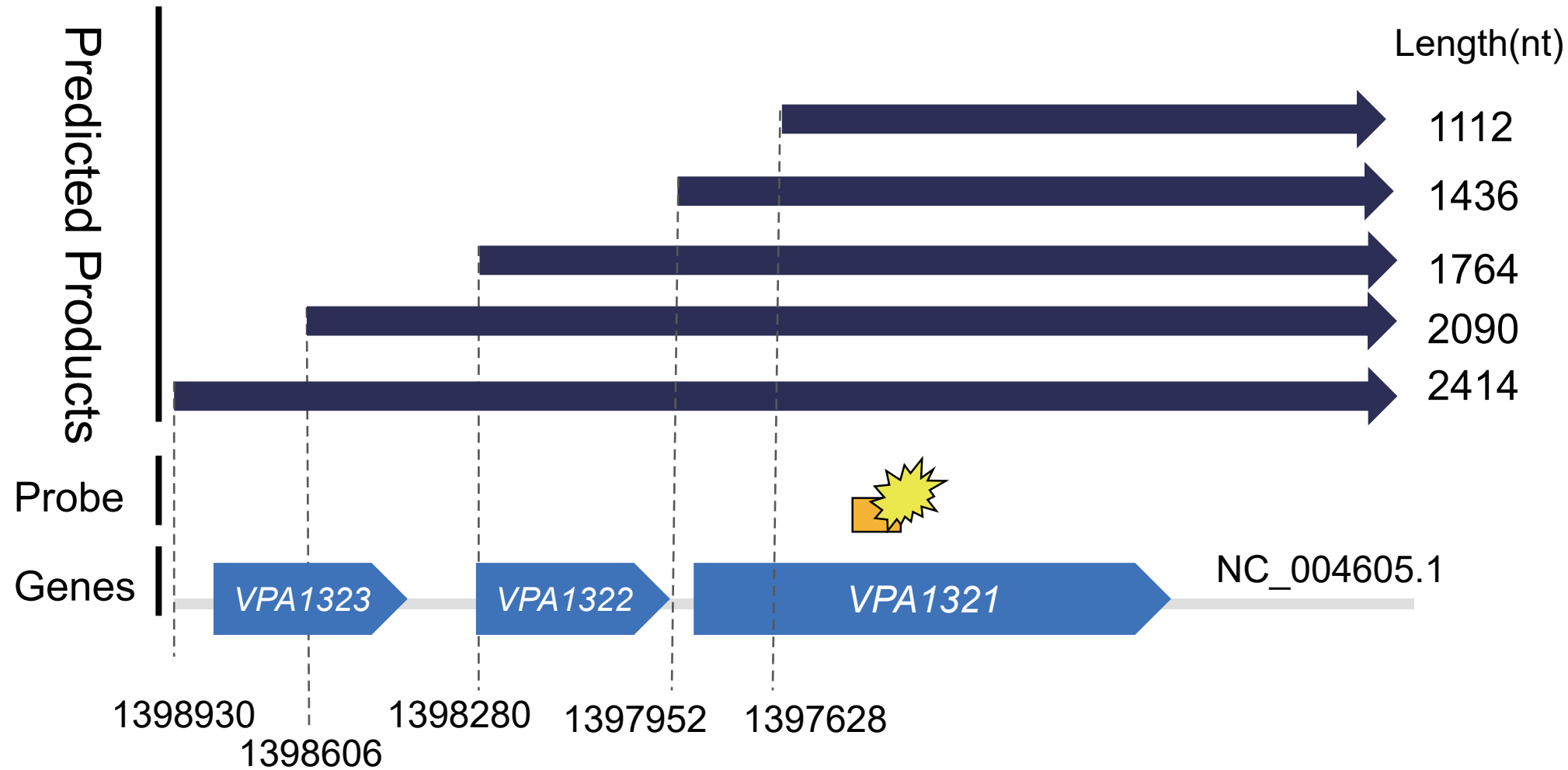

Supplement: FIG S10 [file msystems.00996-21-sf010.pdf]

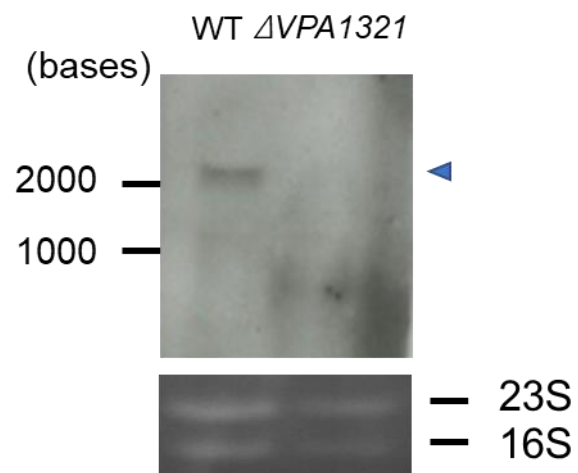

**Supplementary figure 11.** Northern blot analysis with a probe for *VPA1323*.

Supplement: FIG S11 [file msystems.00996-21-sf011.pdf]
