# Supplementary material for: Direct RNA Sequencing Unfolds the Complex Transcriptome of Vibrio parahaemolyticus
Source: mSystems. 2021 Nov 9;6(6):e00996-21. doi: 10.1128/mSystems.00996-21 (PMC8577284; doi:10.1128/mSystems.00996-21)
Supplement: TABLE S4 [file msystems.00996-21-st004.docx]

| **Supplementary table 4.** Comparison of annotated TSSs obtained from literature with detected TSSs. | | | |
| --- | --- | --- | --- |
| **Gene** | **symbol** | **Difference (bp)** | **Study** |
| VP2516 | *OpaR* | -5 | ^39^ |
| VP2762 | *aphA* | 0 | ^39^ |
| VP1698 | *ExsD* | -51 | ^40^ |
| VPA1657 | *psuA* | -10 | ^41^ |
| VPA1658 | *pvsA* | -1 | ^42^ |
| VPA1289 | *CspA* | -8 | ^43^ |
| VPA1446 | *CpsQ* | -9 | ^44^ |
| VPA1445 | *MfbA* | -10 | ^44^ |
| VP2891 | *cadB* | -8 | ^45^ |
| VP2890 | *cadA* | -7 | ^45^ |
| VP1008 | *-* | -10 | ^46^ |
| VP0857 | *feoA* | -7 | ^46^ |
| VP1699 | *exsA* | -9 | ^47^ |
| VP1700 | *exsB* | 6 | ^47^ |
| VPA1332 | *vtrA* | 0 | ^47^ |
| VPA1362 | *vopB2* | 8 | ^47^ |
| VPA1314 | *tdh2* | -2 | ^47^ |
| VPA1027 | *tssD2* | -2 | ^47^ |
| VPA1043 | *tagH2* | -21 | ^47^ |
| VP0820 | *toxR* | -3 | ^48^ |
| VP1687 | *-* | -11 | ^48^ |
| VP1667 | *VopN* | -9 | ^48^ |
